# Dopamine functionalized tannic-acid-templated mesoporous silica nanoparticles as a new sorbent for the efficient removal of Cu^2+^ from aqueous solution

**DOI:** 10.1038/srep45215

**Published:** 2017-03-22

**Authors:** Junkai Gao, Hao Lei, Zhi Han, Qian Shi, Yan Chen, Yanjun Jiang

**Affiliations:** 1 School of Port and Transportation Engineering, Zhejiang Ocean University, Zhoushan 316022, China; 2 School of Chemical Engineering and Technology, Hebei University of Technology, Tianjin, 300130, China

## Abstract

A simple, environmentally friendly and cost-effective nonsurfactant template method was used to synthesize tannic-acid-templated mesoporous silica nanoparticles (TMSNs), and then dopamine functionalized TMSNs (Dop-TMSNs) which was synthesized by a facile and biomimetic coating strategy, was developed as a new sorbent for the removal of Cu^2+^ from aqueous solution. The Dop-TMSNs were thoroughly characterized by SEM, TEM, BET, FT-IR and TGA, and the effects of contact time, initial pH, K^+^ and Na^+^ concentrations, co-existing polyvalent metal ions and adsorption-desorption cycle times on the sorption capacity of Dop-TMSNs were studied. It was demonstrated that the maximum adsorption capacity of Cu^2+^ by Dop-TMSNs was 58.7 mg/g at pH 5.5, and the sorption reached equilibrium within 180 min. Moreover, the K^+^ and Na^+^ concentrations had a very slight influence on the sorption process and the adsorption capacity of the Dop-TMSNs still remained 89.2% after recycling for four times. All the results indicated that the Dop-TMSNs could be utilized as an excellent sorbent for the sequestration of Cu^2+^.

Cu^2+^ is regarded as one of the most harmful heavy metal ions due to its non-biodegradability, high toxicity and bioaccumulation^1^,^2^. There are many sources of Cu^2+^ pollution, which are mostly produced by industries such as chemical manufacturing, paints and pigments, paper and pulp, petroleum refining, metallurgical mining, electroplating and steel-works^1^,^3^,^4^. To eliminate water pollution, the development of efficient methods about removing Cu^2+^ from wastewater becomes highly required.

Currently, the common strategies for removing Cu^2+^ from water include ion exchange, chemical precipitation, adsorption (physi-, chemi- and bio-sorption), catalytic reduction, flotation, membrane filtration, and electrodeposition^5^,^6^,^7^,^8^. Among them, adsorption is considered to be a promising method due to its relatively low cost, efficient, reliable, and easy of operation^1^.

In recent years, mesoporous materials have become a research focus due to their excellent physical and chemical properties. For instance, the mesoporous solar cells (MSCs) have attracted significant attention with the potentialities to lower the cost of solar power^9^. Organic-inorganic hybrid materials can not only combine the individual advantages of organic or inorganic components, but also can overcome their shortcomings^10^. Notably, mesoporous silica nanoparticles (MSNPs) have attracted more and more attention with aims of being utilized as adsorbent materials owing to their biocompatibility, hydrophilic and easily functionalized surface, and high surface area^11^. For instance, a great many organic molecules can be immobilizated on the surface of mesoporous silica because of the existence of high concentration of silano (si-OH) groups^12^. Furthermore, the efficiency of the removal of Cu^2+^ increased remarkably when the mesoporous silica was modified with organic molecules^11^. A number of studies investigated on this topic had been reported^11^,^12^,^13^. Jeong *et al*. synthesized TCPP-SBA-15 with tetrakis(4-carboxyphenyl)porphyrin functionalized mesoporous silica SBA-15, which was systematically studied as an effective material for the removal of Cu^2+^ from aqueous solution^12^,^13^,^14^. Liu *et al*. reported that amino-functionalized SBA-15 showed exceptional binding ability with Cu^2+^ in waste water^15^. Mureseanu *et al*. developed an effective sorbent for the removal of Cu^2+^ from aqueous solution with N-propylsalicylaldimine functionalized SBA-15 (SA-SBA-15)^16^. Da’na *et al*. grafted 3-aminopropyltrimethoxy-silane on the pore walls of SBA-15 for the removal of Cu^2+^ ions^17^.

Presently, there have been many strategies to prepare MSNPs such as Stöber method, co-structure-directing route and using surfactant as templating agents^18^,^19^. The utilization of templated synthesis includes the following steps: (1) the preparation of template; (2) template-directed synthesis of target materials; (3) template removal^20^. However, this synthetic method of MSNPs has several disadvantages as following: (1) the surfactants are expensive and toxic; (2) the calcinations used to remove the surfactants may lead to the reduction of the amount of silanol groups on the surface of MSNPs because of the high-temperature. Hence, a non-toxic, low-cost and especially nonsurfactant templating method that utilized to synthesize MSNPs with interconnected pores is highly required^21^.

Currently, tannic acid (TA), which can be used as a porogen, has attracted great research attention for the reason that it is not only cheap, environmentally friendly and nontoxic but also a nonsurfactant template. Gao and Zharov utilized TA as the template in the preparation of mesoporous silica materials with tunable mesopore sizes (from 6 to 13 nm)^9^,^10^,^11^. Jiang *et al*. synthesized TA-MSNs with TA as the template, and then the TA-MSNs were used as the supports for BCL immobilization^21^.

In addition, the traditional surface modification strategies used to functionalize MSNPs still have several limitation such as low adsorption capacity, complexity of experimental procedure and specific equipments^22^. The utilization of dopamine as a surface modification reagent have attracted great research attention, because it is inexpensive, adhesive, and simple to deposit onto substrates without the need of surface pretreatment^23^. Yang *et al*. developed a Cu^2+^ sorbent of PDA-zeolite with bio-inspired polydopamine modified Natural zeolites, and the adsorption capacity of PDA-zeolite reached to 15.65 mg/g at pH 5.5^1^. Zhang *et al*. synthesized amine-functionalized CNTs via coating CNTs with polydopamine first and then grafting the polyethylene polyamine on the surface of the polydopamine coated CNTs, and the amine-functionalized CNTs showed excellent adsorption efficiency for Cu^2+^ as compared with that of pristine CNTs^22^. Zhang *et al*. prepared an effective sorbent of Fe_3_O_4_/PDA for the adsorption of pollutants from aqueous solution^24^. However, to the best of our knowledge, dopamine modified tannic-acid-templated mesoporous silica nanoparticles has never been used as sorbent for the removal of Cu^2+^ from aqueous solution.

In this work, the mesoporous silica nanoparticles (TMSNs) were prepared utilizing the tannic acid as a nonsurfactant template, and then the dopamine was grafted on the surface of TMSNs to develop a new sorbent of Dop-TMSNs for the removal of Cu^2+^ from aqueous solution. Several experiments were conducted to systematically investigate the influences of contact time, initial pH, K^+^ and Na^+^ concentrations, co-existing polyvalent metal ions and adsorption-desorption cycles on the sorption process. The results demonstrated that Dop-TMSNs exhibited improved performance for the removal of Cu^2+^, and it has great potential for practical applications.

## Materials and Methods

### Materials

Tannic acid and dopamine·HCl were purchased from Sigma-Aldrich. Tetraethoxysilane (TEOS), ammonium hydroxide, Cu(NO_3_)_2_·3H_2_O, Fe(NO_3_)_3_·9H_2_O, Cd(NO_3_)_2_·4H_2_O, Mg(NO_3_)_2_·6H_2_O and Cr(NO_3_)_3_·9H_2_O were purchased from Meryer, China. Ethanol was purchased from Sinopharm Chemical Reagent Co., Ltd. A standard stock solution (1 g/L) of Cu^2+^ was prepared by dissolving Cu(NO_3_)_2_·3H_2_O in deionized water. The desired Cu^2+^ solutions (10~100 mg L^−1^) were prepared by diluting the standard stock solution. All other reagents were of AR grade and used as received.

### Preparation of dopamine-functionalized TMSNs (Dop-TMSNs)

The TMSNs were synthesized as described by Jiang *et al*.^21^. TMSNs were functionalized with dopamine using the post-grafting method. Specifically, 0.4 g of TMSNs were added into 100 mL of 1.0 g/L dopamine solution, which was freshly prepared in phosphate buffer (pH 8.5), and the suspension was stirred for 3 h. Then, the slurry was centrifuged, washed three times with distilled water, and dried at 40 °C in vacuum for 24 h. The as-prepared solid product was denoted as Dop-TMSNs.

### Batch adsorption experiments

In the batch adsorption experiments, an exact amount of Dop-TMSNs were added into 50 mL Cu(NO_3_)_2_·3H_2_O solution in a conical flask, and the mixture was agitated using a mechanical shaker at 200 rev/min at 298 K. The initial pH of solutions was adjusted with 0.1 M HNO_3_ or 0.1 M NaOH. In the equilibrium study, the initial pH of influent solutions was adjusted to 5.5. When the sorption equilibrium was reached, the solid was filtered, and the concentration of Cu^2+^ in the filtrate was measured by a atomic absorption spectrophotometer (361CRT, INESA Analytical Instrument Company Limited, China). The experiments were performed twice, and all the results were reported as average values.

### Adsorption-desorption recycle experiments

The adsorption-desorption recycle experiments were carried out by adding 12 mg of Dop-TMSNs into 20 mL of 50 mg/L Cu(NO_3_)_2_·3H_2_O solution (pH5.5) at 298 K, and the suspension was shaken for 210 min. The solution was then centrifuged at 8000 rpm for 30 minutes, followed by removing 10 mL supernatant for the determination of Cu^2+^ concentration. Subsequently, a certain amount of 0.1 M HNO_3_ solution was added, and then the solution was shaken at 298 K for 210 min to desorb the loaded Cu^2+^ ions, and the final Cu^2+^ concentration was determined. Then, the Dop-TMSNs were recovered and washed with deionized water to reuse in the reaction cycle with fresh Cu(NO_3_)_2_·3H_2_O solution.

### Characterization

The SEM images of Dop-TMSNs were obtained by a scanning electron microscopy (S-4800, Hitachi, Japan). The TEM images were realized by a transmission electron microscopy (JEM-2100F, JEOL, Japan) operating at 200 kV. The surface area measurements of Dop-TMSNs and TMSNs were based on the Brunauer-Emmett-Teller method (BET, BELSORP-max, BEL, Japan), and the pore size distributions were obtained by the Barrett-Joyner-Halenda (BJH) method. The Fourier transform infrared (FT-IR, TENSOR-27, Bruker, Germany) spectra of Dop-TMSNs and TMSNs were recorded using the KBr pellet method. The thermogravimetric analysis of the Dop-TMSNs was measured with a thermogravimetric analyzer (TGA, HCT-1, Beijing henven, China), and the sample was heated at a rate of 10 °C per minute from ambient temperature to 800 °C under a high-purity nitrogen atmosphere.

## Results and Discussion

### Characterization of Dop-TMSNs

**Figure 1:**
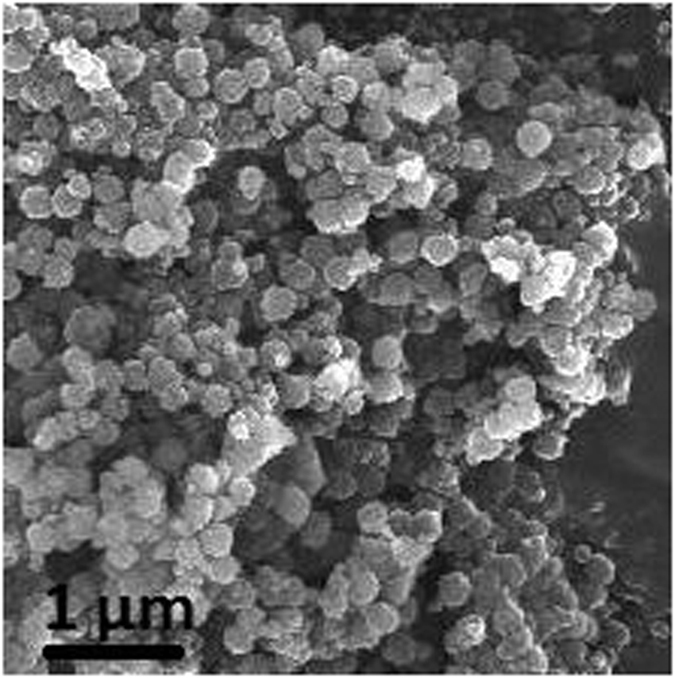
SEM photograph of Dop-TMSNs.

**Figure 2:**
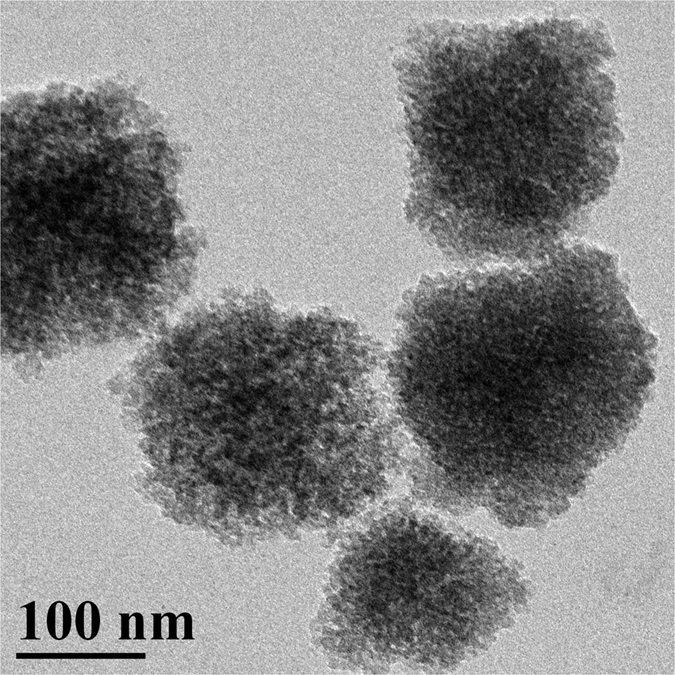
TEM image of Dop-TMSNs.

The SEM and TEM images of Dop-TMSNs are shown in Fig. 1 and Fig. 2. The SEM photograph (Fig. 1) revealed that the Dop-TMSNs were mostly spherical and monodisperse particles, and the average diameter of the particles was about 130–180 nm. The TEM image (Fig. 2) showed that the Dop-TMSNs possessed porous structure with disordered pore arrangement, which was in agreement with the previous reports^11^,^21^.

**Figure 3:**
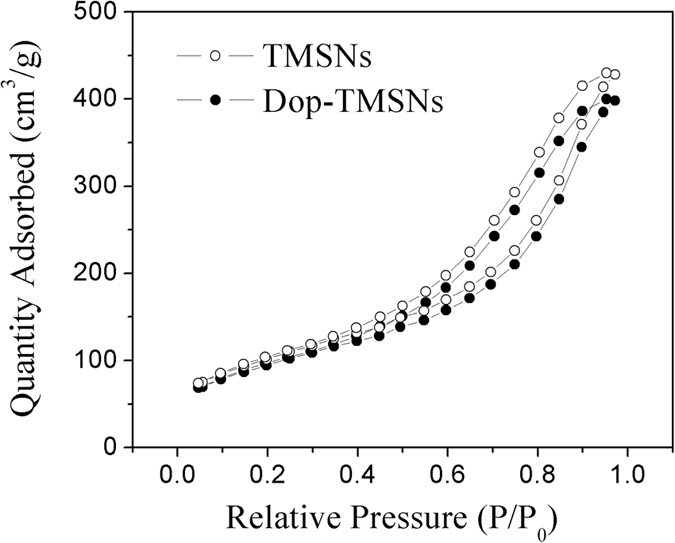
N_2_ adsorption/desorption isotherms of the TMSNs and Dop-TMSNs.

The nitrogen adsorption/desorption isotherms of TMSNs and Dop-TMSNs are shown in Fig. 3. According to the surface area measurements based on the BET method, the surface area of TMSNs was 454 m^2^/g, and the calculated BJH pore size and pore volume were 7.3 nm and 0.72 cm^3^/g, respectively. After modification with dopamine, the surface area, pore size and pore volume of Dop-TMSNs were 396 m^2^/g, 6.9 nm and 0.63 cm^3^/g, respectively. The large pore volume and pore size were beneficial for the Cu^2+^ to enter into the internal structure of Dop-TMSNs, which could enhance the sorption capacity^25^. Compared with the pristine TMSNs, the Dop-TMSNs showed an apparent decrease in the surface area, pore size and pore volume, which indicated that the dopamine was successfully grafted on the surface of TMSNs.

**Figure 4:**
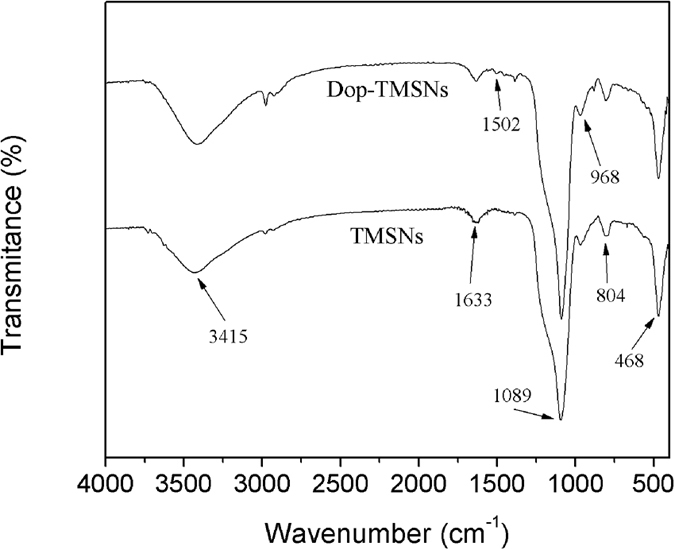
FT-IR spectra of the TMSNs and Dop-TMSNs.

Figure 4 shows the FT-IR spectra of TMSNs and Dop-TMSNs. The peaks at 3415 cm^−1^ and 1633 cm^−1^ were assigned to the stretching and bending vibration of hydroxyl and water, respectively^26^. In all spectra, the typical peaks at 804 cm^−1^ and 468 cm^−1^ corresponded to the symmetric stretching and bending vibration of Si-O-Si, respectively, and the peaks at 1089 cm^−1^ and 968 cm^−1^ were assigned to the asymmetric stretching of Si-O-Si and symmetric stretching of Si-OH, respectively, indicating that the structure of TMSNs was well preserved in the Dop-TMSNs^27^. For the Dop-TMSNs, the peak at 1502 cm^−1^ was belong to the stretching of aromatic rings in the polydopamine, which demonstrated that the dopamine was successfully grafted on the surface of TMSNs^24^.

**Figure 5:**
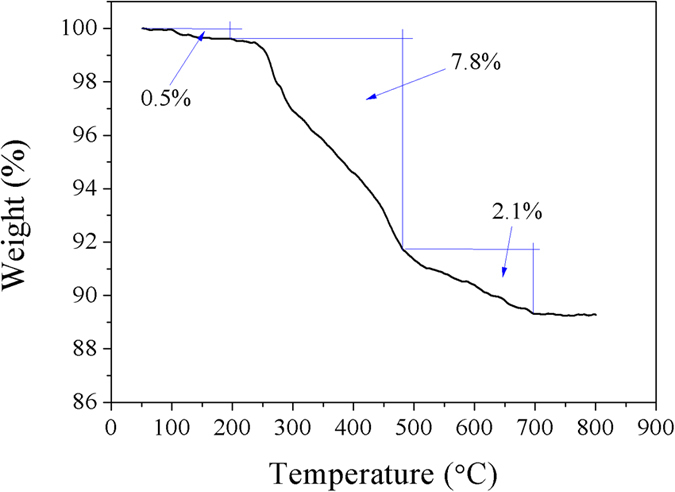
TGA profile of the Dop-TMSNs.

Figure 5 shows the TGA profile for the Dop-TMSNs. It is clear that the weight loss of Dop-TMSNs with the increase of temperature showed three stages. In the first stage, about 0.5% of weight loss occurred from ambient temperature to 190 °C, which was attributed to the volatilization of absorbed water in the pores of Dop-TMSNs. In the second stage, from 190 °C to around 480 °C a weight loss of about 7.8% was ascribed to the decomposition of the polydopamine grafted on the outside surface of the Dop-TMSNs. In the third stage, the weight loss of 2.1% between 480 °C and 690 °C was associated with the pyrolysis of the grafted functional groups of polydopamine inside the pores. According to the above results, the total amount of the grafted functional groups in the Dop-TMSNs was about 9.9%, which further confirmed the successful modification of TMSNs with dopamine.

### Sorption kinetics

**Figure 6:**
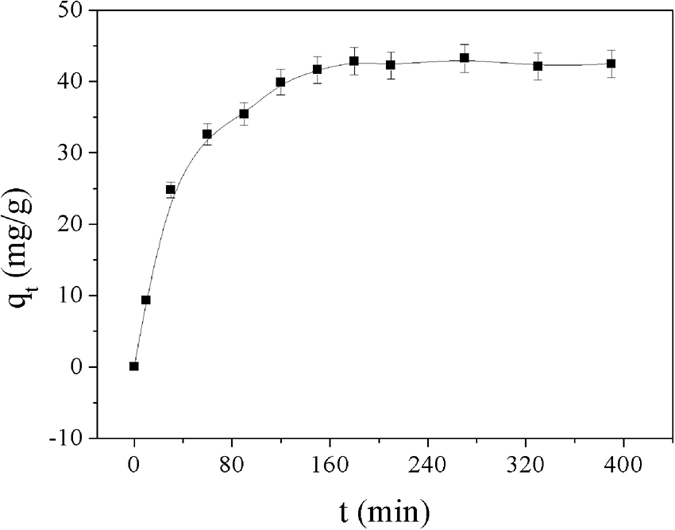
The effect of contact time on the sorption of Cu^2+^ on Dop-TMSNs.

The sorption kinetics were determined with an initial Cu^2+^ concentration of 100 mg/L at 298 K and pH 5.5, and the dosage of Dop-TMSNs was 1.0 g/L. The effect of contact time on the sorption of Cu^2+^ by Dop-TMSNs is shown in Fig. 6. The amount of Cu^2+^ adsorbed on Dop-TMSNs increased with the increase of contact time. It could be observed that the adsorption occurred rapidly within the fist 120 min, and gradually reached equilibrium at 180 min. In the first stage of 0–120 min, the faster adsorption rate could be ascribed to the larger quantity of binding sites on the surface of Dop-TMSNs and the higher Cu^2+^ concentration in the solution. In the second stage of 120–180 min, the amount of Cu^2+^ adsorbed on Dop-TMSNs increased slowly, which could be attributed to that the Cu^2+^ penetrated into the inside pores of Dop-TMSNs needed more energy. Actually, approximate 76.1% of the equilibrium adsorption quantity was obtained within 60 min. To ensure the completeness of adsorption, a contact time of 210 min was used in the subsequent experiments.

The pseudofirst order and pseudosecond order kinetics models, which were shown in Eqs (1) and (2)^28^, were applied to study the specific kinetic parameters of Cu^2+^ adsorpted on Dop-TMSNs.


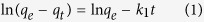



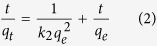


where *q*_*e*_ (mg/g) is the adsorption capacity at equilibrium; *k*_*1*_ (min^−1^) and *k*_*2*_ (g/(mg min)) represent the rate constants of the pseudofirst order adsorption and pseudosecond order adsorption, respectively.

**Figure 7:**
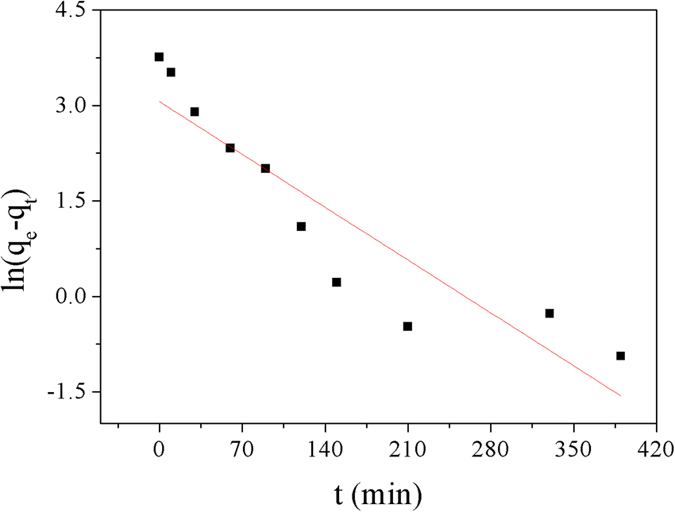
Linearized pseudofirst order kinetic model plot for the Cu^2+^ sorption by Dop-TMSNs.

**Figure 8:**
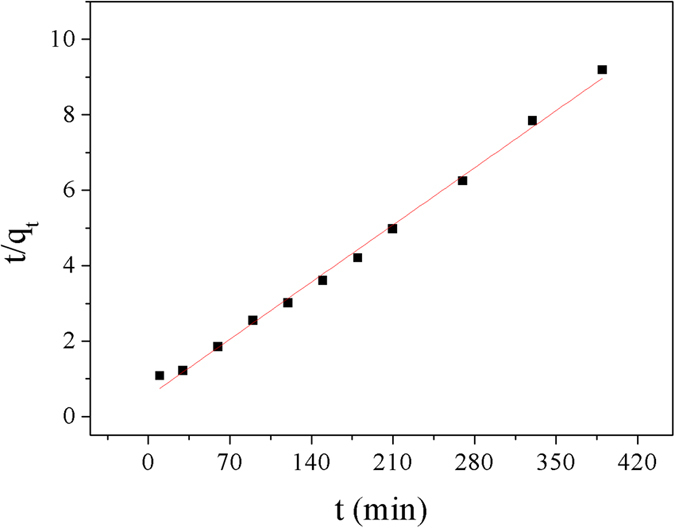
Linearized pseudosecond order kinetic model plot for the Cu^2+^ sorption by Dop-TMSNs.

The values of *k*_*1*_ and *k*_*2*_ could be determined by the slope and intercept of the lines in Figs 7 and 8, respectively, and the results were summarized in Table 1. According to the results, the coefficient (*R*^2^) of the pseudosecond order kinetic equation (*R*^2^ = 0.995) was higher than that of the pseudofirst order kinetic equation (*R*^2^ = 0.827). Furthermore, the *q*_*e*_ value calculated from the pseudosecond order kinetic model, which was 45.4 mg/g, was close to the experimental value of 42.8 mg/g. Hence, the pseudosecond order kinetics model was more suitable for the description of the adsorption kinetics of Cu^2+^ on Dop-TMSNs.

### The effect of initial pH

**Figure 9:**
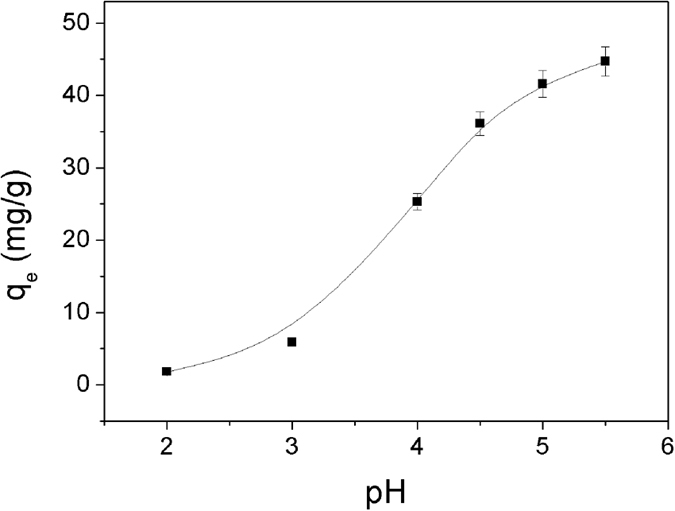
The effect of initial pH on the sorption of Cu^2+^ on Dop-TMSNs.

The initial pH values of the Cu^2+^ solutions have important influence on the adsorption of copper ions^29^,^30^. According to the previous research reports, it was unsuitable for adsorption experiments to be carried out when the pH value of the Cu^2+^ solution was greater than 5.5, for the reason that Cu(OH)_2_ precipitation will be observed at pH above 5.5^12^,^31^. Thus, the adsorption of Cu^2+^ by Dop-TMSNs was investigated by varying the initial pH values of the Cu^2+^ solutions from 2 to 5.5 at 298 K, and the initial Cu^2+^ concentration and Dop-TMSNs dosage were 100 mg/L and 1.0 g/L, respectively. Figure 9 shows the effect of initial pH values on the sorption of Cu^2+^ with Dop-TMSNs. The results indicated that the adsorption capacity of Dop-TMSNs increased with the growth of pH value from 2.0 to 5.5. This phenomenon might be due to the protonation of the functional groups on the surface of Dop-TMSNs at lower pH, which reduces the complexation of the secondary amine group with Cu^2+^ considerably^12^. Thus, the adsorption of Cu^2+^ on Dop-TMSNs was pH-dependent.

### The sorption isotherm

**Figure 10:**
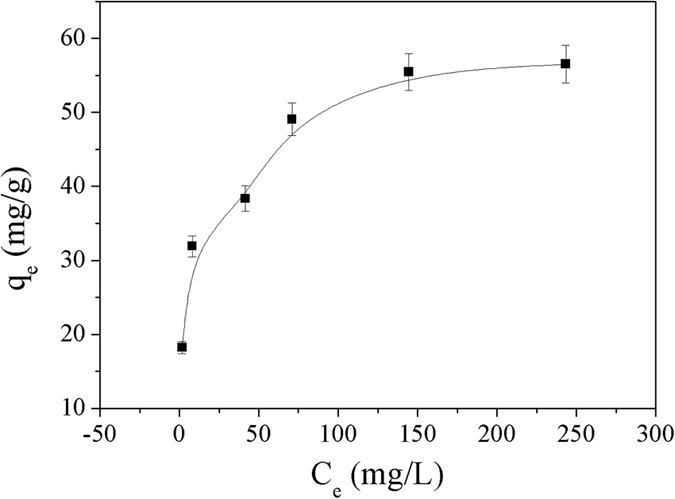
The sorption isotherm of Cu^2+^ on Dop-TMSNs.

The sorption isotherm of Cu^2+^ on Dop-TMSNs was measured by increasing the Cu^2+^ concentration from 20 mg/L to 300 mg/L at 298 K and pH 5.5, and the Dop-TMSNs dosage was 1.0 g/L. As shown in Fig. 10, with the increase of equilibrium Cu^2+^ concentration, the adsorption capacity of Dop-TMSNs for Cu^2+^ increased and the maximum adsorption capacity was 58.7 mg/g. The superior adsorption capacity was mainly attributed to that the Dop-TMSNs had large surface areas and the functional groups on the surface of Dop-TMSNs had excellent Cu^2+^ chelation ability.

Additionally, the Cu^2+^ sorption capacity of Dop-TMSNs was compared with other adsorbents, and the results are shown in Table 2. Although the adsorption capacity of Dop-TMSNs was not the largest one, the synthetic method of Dop-TMSNs used in this work was simple, economic, environmentally friendly and nontoxic. Thus, the Dop-TMSNs had great potential for widespread practical applications.

To study the adsorption behaviour between the Cu^2+^ and Dop-TMSNs, Langmuir and Freundlich isotherms were used to analyze the equilibrium adsorption data^27^,^28^,^29^,^30^,^31^,^32^. Linear equations of Langmuir and Freundlich models are shown in Eqs (3) and (4)^33^,^34^:


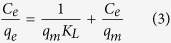



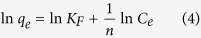


where *q*_*m*_ (mg/g) and *K*_*L*_ are the maximum adsorption capacity for fitting and a constant related to the free energy of adsorption, respectively. *K*_*F*_ and *n* are the constants for Freudlich model.

**Figure 11:**
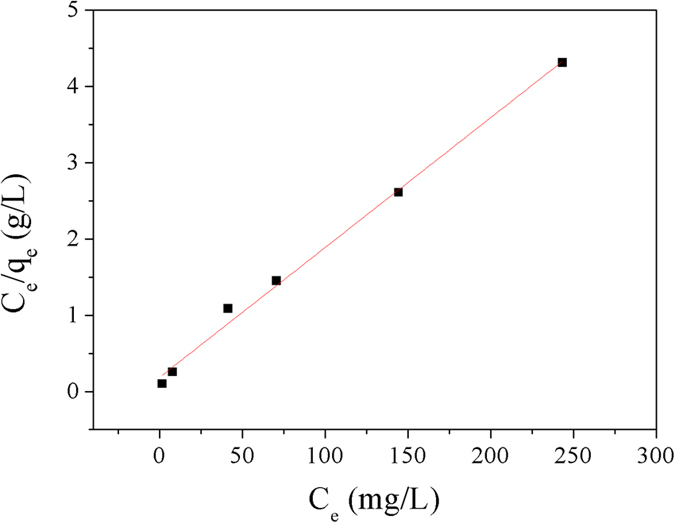
Linearized Langmuir model plot for the Cu^2+^ sorption by Dop-TMSNs.

**Figure 12:**
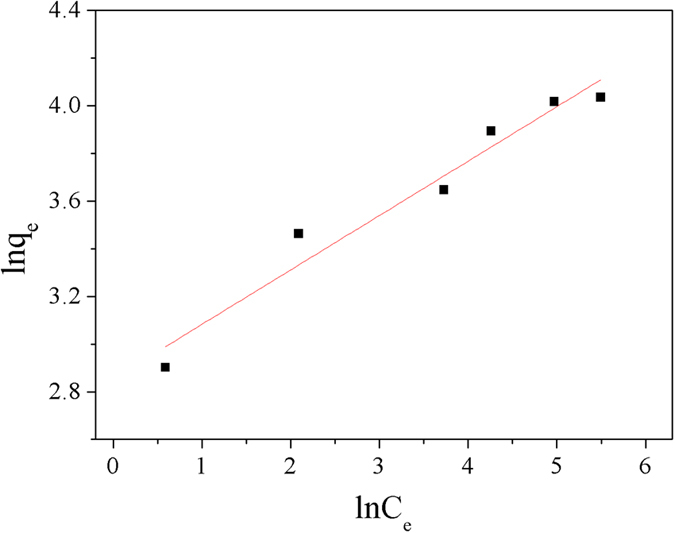
Linearized Freundlich model plot for the Cu^2+^ sorption by Dop-TMSNs.

In agreement with Eqs. (3) and (4), two straight lines were shown in Fig. 11 and 12, respectively. The regression coefficient (*R*^2^) in Table 3 indicated that the sorption data of Cu^2+^ on Dop-TMSNs fitted the Langmuir model (*R*^2^ = 0.994) better than the Freundlich model (*R*^2^ = 0.948). Additionally, the calculated Langmuir adsorption capacity (58.7 mg/g) was close to the experimental adsorption capacity (55.6 mg/g). Therefore, the Langmuir model could properly describe the observed adsorption process, which indicated that the adsorption of Cu^2+^ might take place at homogeneous binding sites on the surface of Dop-TMSNs and formed a monolayer^1^,^35^.

**Figure 13:**
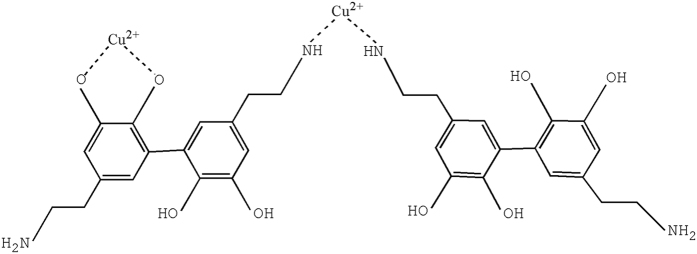
The proposed mechanism of polydopamine interaction with Cu^2+^.

The phenolic groups in polydopamine had chelation ability with a variety of metals^36^, therefore, bidentate chelating bonding in which two oxygen atoms bound to a copper might be one of the mechanisms of polydopamine on the surface of Dop-TMSNs interaction with Cu^2+^ ions, as shown in Fig. 13. Additionally, Cu^2+^ is one of the borderline metals with ambivalent properties that possesses favourable affinity with amine groups^37^, thus the amino ligands in the polydopamine could coordinate with the Cu^2+^ ions, as shown in Fig. 13.

### The effect of K^+^ and Na^+^ concentrations

**Figure 14:**
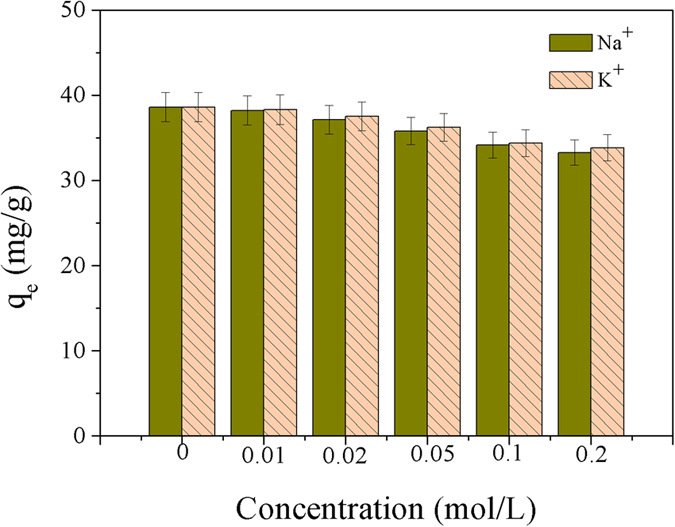
The effect of K^+^ and Na^+^ concentrations on the sorption of Cu^2+^ on Dop-TMSNs.

K^+^ and Na^+^ concentrations are significant factors influencing the sorption of Cu^2+^. To study the effect of K^+^ and Na^+^ concentrations on the Cu^2+^ sorption by Dop-TMSNs, the experiments were carried out in the presence of KNO_3_ or NaNO_3_ with concentrations varying from 0.01 to 0.2 mol/L at 298 K and pH 5.5, and the initial Cu^2+^ concentration and Dop-TMSNs dosage were 50 mg/L and 0.6 g/L, respectively. The results are shown in Fig. 14. It could be seen that the presence of K^+^ or Na^+^ had a very slight influence on the adsorption capacity of Dop-TMSNs for Cu^2+^, which might be attributed to that the surface functional groups on Dop-TMSNs had stronger chelation capability with Cu^2+^ than with K^+^ or Na^+^. Furthermore, the possible explanation for the decline of Cu^2+^ sorption capacity of Dop-TMSNs might be that the increase of ionic strength resulted in the decrease of the activity of Cu^2+^ ions, and the K^+^ or Na^+^ ions also had competition with Cu^2+^ ions for the active sites on Dop-TMSNs^25^.

### The effect of co-existing polyvalent metal ions

**Figure 15:**
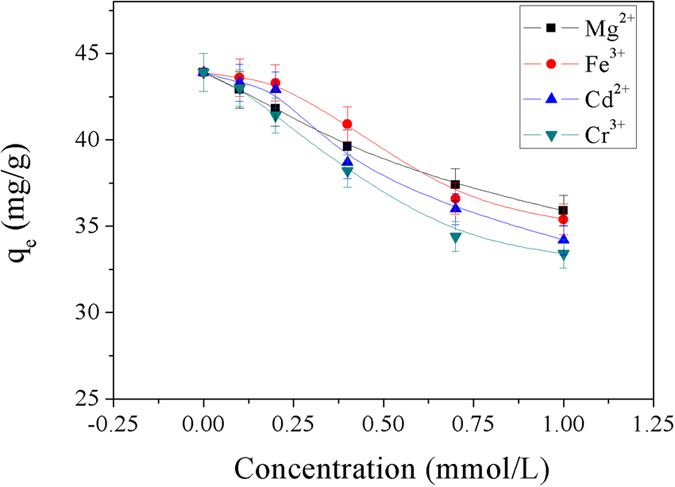
The effect of co-existing polyvalent metal ions on the sorption of Cu^2+^ on Dop-TMSNs.

To study the effect of co-existing polyvalent metal ions on the Cu^2+^ sorption by Dop-TMSNs, the experiments were carried out at 298 K and pH 5.5 in the presence of Fe(NO_3_)_3_, Cd(NO_3_)_2_, Mg(NO_3_)_2_ or Cr(NO_3_)_3_ with concentrations varying from 0.1 to 1 mmol/L, respectively, and the initial Cu^2+^ concentration and Dop-TMSNs dosage were 1 mmol/L and 0.6 g/L, respectively. As illustrated in Fig. 15, it was obvious that the presence of polyvalent metal ions had a very slight influence on the adsorption capacity as the concentration of the metal ions was below 0.2 mmol/L. With increasing the concentrations of the metal ions, the adsorption capacity decreased, which might be ascribed to the competition between the polyvalent metal ions and Cu^2+^ ions for the active adsorption sites^38^. However, the co-existing polyvalent metal ions had insignificant influence on the adsorption of Cu^2+^ ions on Dop-TMSNs. Additionally, among the polyvalent metal ions, the Cr^3+^ ions had the greatest influence on the adsorption capacity of Dop-TMSNs for Cu^2+^, which might be attributed to that the Cr^3+^ ions had stronger affinity with the adsorption sites than the other three metal ions.

### Desorption and reusability

**Figure 16:**
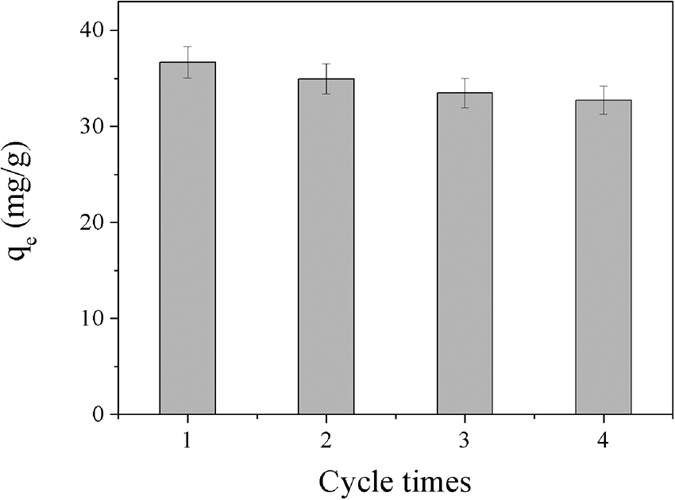
The reusability of Dop-TMSNs for four cycles.

The regeneration and reuse of Dop-TMSNs can make the adsorption process cost-effective in the large-scale application. To test the reusability of Dop-TMSNs, the adsorption-desorption experiments were carried out with the Dop-TMSNs dosage of 0.6 g/L and the initial Cu^2+^ concentration of 50 mg/L at 298 K and pH 5.5. As illustrated in Fig. 16, the Dop-TMSNs could still remain 89.2% of its initial adsorption capacity after four adsorption-desorption recycle experiments. The decrease of adsorption capacity of Dop-TMSNs might be ascribed to the loss of the sorbent or the irreversible occupation of part adsorption sites^25^. These results suggested that the reusability of Dop-TMSNs was superior, and it could be used as a recyclable and efficient adsorbent for the removal of Cu^2+^.

### Conclusions

In conclusion, a dopamine functionalized tannic-acid-templated mesoporous silica nanoparticles (Dop-TMSNs) for the removal of Cu^2+^ was synthesized by a facile, simple, environmentally friendly and cost-effective method. The maximum adsorption capacity of Dop-TMSNs was 58.7 mg/g at 298 K and pH 5.5. The adsorption equilibrium was reached with 180 min, and the adsorption kinetics could be described well by the pseudosecond order kinetics model. The sorption isotherm parameters were fited well with the Langmuir model, and the adsorption capacity of Dop-TMSNs could still remain 89.2% after recycling for four times. Additionally, the Cu^2+^ adsorption by Dop-TMSNs was pH dependent, and the influence of K^+^ and Na^+^ concentrations was very weak. Taken together, the Dop-TMSNs had great potential to be utilized as Cu^2+^ adsorbent in practical applications.

## Additional Information

**How to cite this article**: Gao, J. *et al*. Dopamine functionalized tannic-acid-templated mesoporous silica nanoparticles as a new sorbent for the efficient removal of Cu^2+^ from aqueous solution. *Sci. Rep.* **7**, 45215; doi: 10.1038/srep45215 (2017).

**Publisher's note:** Springer Nature remains neutral with regard to jurisdictional claims in published maps and institutional affiliations.

## Figures and Tables

**Table 1:** Kinetic parameters of Cu^2+^ adsorbed on Dop-TMSNs.

Pseudofirst order			Pseudosecond order		
*k*_*1*_ (min^−1^)	*q*_*e*_ (mg/g)	*R*^2^	*k*_*2*_(g/(mg min))	*q*_*e*_ (mg/g)	*R*^2^
0.012	21.3	0.827	0.001	45.4	0.995

**Table 2:** Comparison of Dop-TMSNs with other adsorbents.

Adsorbent	Sorption capacity (mg/g)	References
Dop-TMSNs	58.7	This work
MPAL	32.19	39
SBA-TACN	42.6	12
CCHBs	63.7	40
Polydopamine nanoparticles	34.4	41
Chitosan	85.21	42
Polydopamine coated zeolites	28.58	1
Pristine natural zeolite	14.93	1
AMS	53.3	13

**Table 3:** Adsorption isotherm parameters of Cu^2+^ adsorbed on Dop-TMSNs.

Model parameters	Langmuir			Freundlich		
	*K*_*L*_	*q*_*m*_ (mg/g)	*R*^2^	*K*_*F*_	*n*	*R*^2^
Value	0.092	58.7	0.994	17.4	4.38	0.948

## References

[b1] Yu Y., Shapter J. G., Popelka-Filcoff R., Bennett J. W. & Ellis A. V. Copper removal using bio-inspired polydopamine coated natural zeolites. J. Hazard. Mate. 273, 174–82 (2014). 10.1016/j.jhazmat.2014.03.048 24731937

[b2] Hao S., Zhong Y., Pepe F. & Zhu W. Adsorption of Pb^2+^ and Cu^2+^ on anionic surfactant-templated amino-functionalized mesoporous silicas. Chem. Eng. J. 189–190, 160–167 (2012).

[b3] Anirudhan T. S. & Rijith S. Physicochem. Glutaraldehyde cross-linked epoxyaminated chitosanas an adsorbent for the removal and recovery of copper(II) from aqueous media. Eng. Aspects. 351, 52–59 (2009).

[b4] Chiron N., Guilet R. & Deydier E. Adsorption of Cu(II) and Pb(II) onto a grafted silica: isotherms and kinetic models. Water Res. 37, 3079–3086 (2003). 14509694 10.1016/S0043-1354(03)00156-8

[b5] Chang J. H., Ellis A. V., Tung C. H. & Huang W. C. Copper cation transport and scaling of ionic exchange membranes using electrodialysis under electroconvection conditions. J. Membr. Sci. 361, 56–62 (2010).

[b6] Ahmaruzzaman M. Industrial wastes as low-cost potential adsorbents for thetreatment of wastewater laden with heavy metals. Adv. Colloid Interface Sci. 166, 36–59 (2011). 21669401 10.1016/j.cis.2011.04.005

[b7] Madadrang C. J. . Adsorption behavior of EDTA-graphene oxide for Pb(II) removal. ACS Appl. Mater. Interface. 4, 1186–1193 (2012). 10.1021/am201645g 22304446

[b8] Fu F. & Wang Q. Removal of heavy metal ions from wastewaters: a review. J. Environ. Manage. 92, 407–418 (2011). 21138785 10.1016/j.jenvman.2010.11.011

[b9] Erwin W. R. . Light trapping in mesoporous solar cells with plasmonic nanostructures. Energy Environ. Sci. 9, 1577–1601 (2016).

[b10] Chen Y. & Shi J. L. Chemistry of Mesoporous Organosilica in Nanotechnology: Molecularly Organic-Inorganic Hybridization into Frameworks. Adv. Mater. 28, 3235–3272 (2016). 26936391 10.1002/adma.201505147

[b11] Gao Z. & Zharov I. Large pore mesoporous silica nanoparticles by templating with a nonsurfactant molecule, tannic acid. Chem. Mater. 26, 2030–2037 (2014).

[b12] Tapaswi P. K., Moorthy M. S., Park S. S. & Ha C. S. Fast, selective adsorption of Cu^2+^ from aqueous mixed metal ions solution using 1,4,7-triazacyclononane modified SBA-15 silica adsorbent (SBA-TACN). J. Solid. State. Chem. 211, 191–199 (2014).

[b13] Hu Z., Zhang X., Zhang D. & Wang J. X. Adsorption of Cu^2+^ on amine-functionalized mesoporous silica brackets. Water Air Soil Poll. 223, 2743–2749 (2012).

[b14] Jeong E. Y., Ansari M. B., Mo Y. H. & Park S. E. Removal of Cu(II) from water by tetrakis(4-carboxyphenyl) porphyrin-functionalized mesoporous silica. J. Hazard. Mater. 185, 1311–1317 (2011). 21055872 10.1016/j.jhazmat.2010.10.047

[b15] Liu A. M., Hidajat K., Kawi S. & Zhao D. Y. A new class of hybrid mesoporous materials with functionalized organic monolayers for selective adsorption of heavy metal ions. Chem. Commun. 13, 1145–1146 (2000).

[b16] Mureseanu M. . Modified SBA-15 mesoporous silica for heavy metal ions remediation. Chemosphere 73, 1499 (2008). 18760443 10.1016/j.chemosphere.2008.07.039

[b17] Da’na E. & Sayari A. Adsorption of copper on amine-functionalized SBA-15 prepared byco-condensation: equilibrium properties. Chem. Eng. J. 166, 445–453 (2011).

[b18] Eisuke Y. & Kazuyuki K. Mesoporous Silica Nanoparticles. Bull. Chem. Soc. Jpn. 89, 501–539 (2016).

[b19] Huang Z. H. & Che S. A. Fabrication of Mesostructured Silica Materials through Co-Structure-Directing Route. Bull. Chem. Soc. Jpn. 88, 617–632 (2015).

[b20] Malgras V. . Templated Synthesis for Nanoarchitectured Porous Materials. Bull. Chem. Soc. Jpn. 88, 1171–1200 (2015).

[b21] Jiang Y. . Improved performance of lipase immobilized on tannic acid-templated mesoporous silica nanoparticles. Appl. Biochem. Biotech. 179, 1155–1169 (2016). 10.1007/s12010-016-2056-1 27011329

[b22] Zhang X. . Preparation of amine functionalized carbon nanotubes via a bioinspired strategy and their application in Cu^2+^ removal. Appl. Surf. Sci. 343, 19–27 (2015).

[b23] Lee M. . Water detoxification by a substrate-bound catecholamine adsorbent. Chem. Plus. Chem. 77, 987–990 (2012). 23745174 10.1002/cplu.201200209 PMC3670806

[b24] Zhang S. . Mussel-inspired polydopamine biopolymer decorated with magnetic nanoparticles for multiple pollutants removal. J. Hazard. Mater. 270, 27–34 (2014). 24525161 10.1016/j.jhazmat.2014.01.039

[b25] Chen Y., Gao J. K., Wen X. F. & Wu W. F. Efficient removal of cadmium using facile functionalized of mesoporous silica via a biomimetic coating. Rsc. Adv. 6, 18340–18347 (2016).

[b26] Feng L. L. . The shape-stabilized phase change materials composed of polyethylene glycol and various mesoporous matrices (AC, SBA-15 and MCM-41). Sol. Energ. Mat. Sol. C. 95, 3550–3556 (2011).

[b27] Gao J. K., Hou L. A., Zhang G. H. & Gu P. Facile functionalized of SBA-15 via a biomimetic coating and its application in efficient removal of uranium ions from aqueous solution. J. Hazard. Mater. 286, 325–333 (2015). 25590826 10.1016/j.jhazmat.2014.12.061

[b28] Wang G., Wang X. & Chai X. Adsorption of uranium(VI) from aqueous solution on calcined and acid-activated kaolin. Appl. Clay. Sci. 47, 448–451 (2010).

[b29] Ge F., Li M. M., Ye H. & Zhao B. X. Effective removal of heavy metal ions Cd^2+^, Zn^2+^, Pb^2+^, Cu^2+^ from aqueous solution by polymer-modified magnetic nanoparticles, J. Hazard. Mater. 211–212, 366–372 (2012). 10.1016/j.jhazmat.2011.12.013 22209322

[b30] Tang W. W. . Impact of humic/fulvic acid on the removal of heavy metals from aqueous solutions using nanomaterials: a review. Sci. Total Environ. 468, 1014–1027 (2014). 24095965 10.1016/j.scitotenv.2013.09.044

[b31] Lee C. I., Yang W. F. & Chiou C. S. Utilization of water clarifier sludge for copper removal in a liquid fluidized-bed reactor. J. Hazard. Mater. 129, 58–63 (2006). 16309828 10.1016/j.jhazmat.2005.06.045

[b32] Ho Y. S. Selection of optimum sorption isotherm, Carbon 42, 2115–2116 (2004).

[b33] Langmuir I. The adsorption of gases on plane surfaces of glass, mica and platinum. J. Am. Chem. Soc. 40, 1361–1403 (1918).

[b34] Freundlich H. M. F. Over the adsorption in solution. J. Phys. Chem. A 57, 385–471 (1906).

[b35] Baba Y., Ohe K., Kawasaki Y. & Kolev S. D. Adsorption of mercury(II) from hydrochloric acid solutions on glycidylmethacrylate-divinylbenzene microspheres containing amino groups. React. Funct. Polym. 66, 1158–1164 (2006).

[b36] Guo J. . Engineering multifunctional capsules through the assembly of metal-phenolic networks. Angew. Chem. 53, 5546–5551 (2014). 24700671 10.1002/anie.201311136

[b37] Shahbazi A., Younesi H. & Badiei A. Functionalized SBA-15 mesoporous silica by melamine-based dendrimer amines for adsorptive characteristics of Pb(II), Cu(II) and Cd(II) heavy metal ions in batch and fixed bed column. Chem. Eng. J. 168, 505–518 (2011).

[b38] Zhang X. . Efficient removal and highly selective adsorption of Hg^2+^ by poly dopamine nanospheres with total recycle capacity. Appl. Surf. Sci. 314, 166–173 (2014).

[b39] Han J., Liang X., Xu Y. & Xu Y. Removal of Cu^2+^ from aqueous solution by adsorption onto mercapto functionalized palygorskite. J. Ind. Eng. Chem. 23, 307–315 (2015).

[b40] Wang J. . Collagen/cellulose hydrogel beads reconstituted from ionic liquid solution for Cu(II) adsorption Carbohydr. Polymer 98, 736–743 (2013). 10.1016/j.carbpol.2013.06.001 23987406

[b41] Wang S., Li H. & Xu L. Application of zeolite MCM-22 for basic dye removal from wastewater. J. Colloid Interf. Sci. 295, 71–78 (2006). 10.1016/j.jcis.2005.08.006 16143340

[b42] Kołody´ nskaz D. Chitosan as an effective low-cost sorbent of heavy metal complexes with the polyaspartic acid. Chem. Eng. J. 173, 520–529 (2011).

